# Simple Nanochannel-Modified Electrode for Sensitive Detection of Alkaline Phosphatase Through Electrochemiluminescence Signal Quenching by Enzymatic Reaction

**DOI:** 10.3390/bios15060377

**Published:** 2025-06-11

**Authors:** Tianjun Ma, Xuan Luo, Fengna Xi, Nuo Yang

**Affiliations:** 1 The First Affiliated Hospital of Guangxi Medical University, Nanning 530021, China; mtj.2008@sr.gxmu.edu.cn; 2 School of Chemistry and Chemical Engineering, Zhejiang Sci-Tech University, Hangzhou 310018, China; 202020104138@mails.zstu.edu.cn

**Keywords:** alkaline phosphatase, electrochemiluminescence, homogeneous sensing, nanochannel-modified electrode, signal quenching

## Abstract

Development of sensitive and convenient alkaline phosphatase (ALP) detection methods is of great significance for food analysis, biomedical applications, and clinical tests. In this work, a sensitive detection method for ALP was established based on nanochannel-modified electrodes, where the electrochemical luminescence (ECL) signal was quenched by the enzymatic reaction product. Vertically ordered mesoporous silica film (VMSF) was rapidly grown on low-cost ITO via the electrochemically assisted self-assembly (EASA) method. The resulting modified electrode (VMSF/ITO) exhibited a uniform and ordered nanochannel structure with nanochannel diameter of 2–3 nm and charge-selective permeability, enabling the enrichment of cationic ECL emitter tris(2,2′-bipyridyl)ruthenium(II) (Ru(bpy)_3_^2+^). Compared to the ITO electrode, VMSF/ITO increased the ECL signal by 60 times. In the presence of ALP, it catalyzes the hydrolysis of its substrate, disodium phenyl phosphate hydrate (DPP), generating phenol (Phe), which quenched the ECL signal of Ru(bpy)_3_^2^^+^ and the co-reactant N,N-Dipropyl-1-propanamine (TPA). Based on this principle, ECL detection of ALP can be achieved. The linear detection range for ALP was 0.01 U/L to 30 U/L, with a limit of detection (LOD) of 0.008 U/L. The sensor exhibited high selectivity. Combined with the anti-contamination and anti-interference capabilities of VMSF, the constructed sensor enabled the detection of ALP levels in milk samples.

## 1. Introduction

Alkaline phosphatase (ALP) is a widely distributed enzyme found in various mammalian tissues (e.g., bone, liver, placenta, intestines, and kidneys) [1,2]. This enzyme has a dimeric structure, composed of two similar monomers. Its active site contains five cysteine residues, two zinc ions, and one magnesium ion. This metal coordination structure imparts high catalytic efficiency, enabling the enzyme to dephosphorylate a variety of substrates, including carbohydrates, proteins, and nucleic acids [3,4,5]. In dairy quality control, ALP is a key biological indicator due to its remarkable thermal stability. Specifically, after pasteurizing milk at 71.6 °C for 15 s, a negative ALP activity test indicates the complete inactivation of heat-resistant pathogens, such as *Mycobacterium tuberculosis* and *Listeria monocytogenes* [6,7]. Consequently, strict dairy safety standards have been established in Europe and the U.S., with the threshold for ALP activity in pasteurized milk set below 0.35 U/L [8]. In clinical diagnostics, ALP is an important serum biomarker. The normal range of ALP in serum is 50–135 U/L for females and 45–125 U/L for males [9,10]. Abnormal serum ALP levels can indicate conditions like bone metabolism disorders, hepatobiliary diseases (e.g., obstructive jaundice, liver cancer), malignant tumors (e.g., breast cancer, prostate cancer), and diabetes [11,12]. This responsive concentration characteristic makes ALP a core indicator in routine clinical tests [13,14]. Additionally, due to its excellent enzymatic properties including broad substrate specificity, high catalytic efficiency, and stable physicochemical characteristics, ALP has been developed as a signal amplification element in enzyme-linked immunosorbent assay (ELISA) systems. By coupling with specific antibodies, the ALP labeling system amplifies the detection signal, significantly enhancing sensitivity [15]. Therefore, the development of sensitive and convenient ALP detection methods is of great significance.

Currently, various analytical methods, such as chromatography [16], fluorescence sensing [17], surface-enhanced Raman scattering (SERS) [18], and colorimetric detection [19], are used for ALP detection. While these methods often rely on large, expensive instruments and specialized personnel resulting from complex sample pretreatment steps like derivatization and enrichment. The detection process is typically time-consuming and involves large sample volumes (usually in milliliters). Additionally, matrix effects and non-specific adsorption can lead to interference, affecting detection reliability. Electrochemiluminescence (ECL), which generates controllable chemiluminescence via electrochemical excitation without requiring an external light source, combines the benefits of simple electrochemical operations, fast response, and a wide chemiluminescence linear range [20,21,22]. These advantages have made ECL widely used in food analysis, environmental detection, and clinical diagnostics [23,24,25,26]. Recent advancements include novel ECL emitters such as lumino-modified gold nanoclusters (lum-AuNCs) [27], CdSe quantum dots with dual stabilizers [28], and CdS−Ru complexes [29]. While these materials show enhanced ECL performance, their synthesis involves time-consuming multi-step surface modification or self-assembly. In classical ECL systems, ruthenium(II) polypyridine complexes (e.g., Ru(bpy)_3_^2+^) are widely used emitters due to their high ECL quantum yield, millisecond response kinetics, and wide pH adaptability [30,31,32]. However, current methods require micromolar concentrations (e.g., 100 μM) of ruthenium complexes to maintain stable signals, resulting in reagent costs of USD 1.5–3.0 per test. Reducing Ru(bpy)_3_^2+^ consumption could further promote ECL applications in bioanalysis. Furthermore, components in complex matrices (e.g., proteins and lipids) can cause non-specific adsorption, significantly affecting detection accuracy and reproducibility. Thus, developing simple, sensitive, and interference-resistant ECL sensing strategies with low consumption of Ru(bpy)_3_^2+^ remains challenging.

Vertically ordered mesoporous silica film (VMSF) is a nanostructured thin film with vertically aligned mesoporous nanochannels [33,34,35]. Its unique structural features provide two core functionalities including molecular sieving and charge selectivity. The ultra-small nanochannels (2–3 nm) of VMSF effectively block large biomolecules, such as proteins and liposomes, from entering the nanochannels and contaminating the electrode surface, thus reducing the impact of biological matrices on detection [36,37,38,39]. The abundant silanol groups (Si-OH, p*K*_a_~2) on the surface of VMSF ionize under normal conditions to form a negatively charged surface, which can efficiently enrich positively charged molecules, such as the ECL emitter Ru(bpy)_3_^2+^, via electrostatic interactions, thereby enhancing detection sensitivity [40,41,42,43,44]. This synergistic mechanism based on sieving and enrichment enables VMSF-modified electrodes to exhibit excellent resistance to matrix interference and sensitive detection [45,46,47]. Furthermore, VMSF can be easily modified onto the surface of indium tin oxide (ITO) [48]. ITO electrodes offer superior performance (sheet resistance < 15 Ω/sq, transmittance > 85%), low cost (about 1/20th the price of gold electrodes), and good chemical stability. Compared to the commonly used carbon electrodes (e.g., glass carbon electrode-GCE) [49,50], the surface of ITO is rich in hydroxyl groups (–OH), which can form Si–O–In covalent bonds with the silanol groups of VMSF through dehydration condensation, allowing stable modification of VMSF on the ITO electrode surface without the need for silane coupling agents [51]. This bonding strategy not only simplifies the preparation process but also ensures high mechanical stability at the film-electrode interface. Therefore, VMSF-modified ITO electrodes show great potential for sensitive and interference-resistant ECL sensing of ALP.

**Figure 1:**
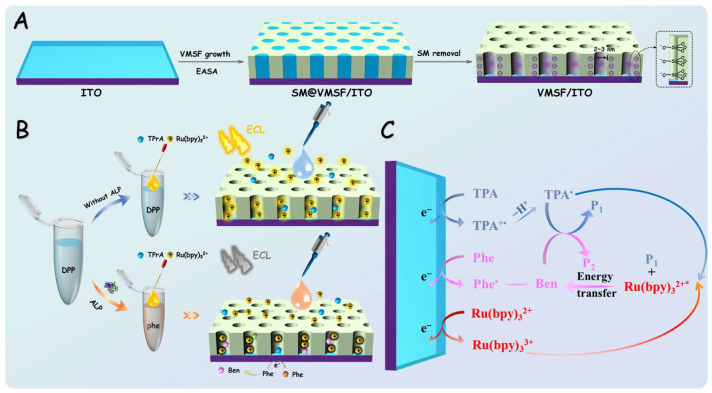
Schematic illustration of the preparation of VMSF/ITO electrode (**A**), ALP detection using the enzymatic reaction and VMSF/ITO electrode (**B**), and (**C**) the ECL quenching mechanism of Ru(bpy)_3_^2^^+^-TPA by Phe produced through the ALP-catalyzed reaction.

This work presented a simple and highly sensitive ECL detection method for ALP. As illustrated in Figure 1, VMSF-modified ITO electrodes were simply prepared and the positively charged ECL emitter Ru(bpy)_3_^2+^ was enriched in the nanochannels. In the presence of ALP, its substrate disodium phenyl phosphate (DPP) was specifically hydrolyzed to generate phenol (Phe) [52], which quenched the ECL signal of the Ru(bpy)_3_^2+^ and the co-reactant tripropylamine (TPA) system [53]. Based on this mechanism, combined with the anti-interference and anti-fouling capabilities of VMSF/ITO, sensitive detection of ALP in milk and human serum samples was achieved. The constructed sensor offers advantages including simple electrode preparation, low consumption of ECL emitters, and high detection sensitivity.

## 2. Materials and Methods

### 2.1. Chemicals and Materials

Tetraethyl orthosilicate (TEOS), cetyltrimethylammonium bromide (CTAB), sodium hydrogen phosphate dodecahydrate (Na_2_HPO_4_·12H_2_O), potassium ferrocyanide (K_3_[Fe(CN)_6_]), potassium hydrogen phthalate (KHP), alkaline phosphatase (ALP), glucose dehydrogenase (GDH), alcohol dehydrogenase (ADH), horseradish peroxidase (HRP), and glucose oxidase (GOx) were purchased from Aladdin Biochemical Technology Co., Ltd. (Shanghai, China). Disodium phenyl phosphate (DPP), tripropylamine (TPA), and sodium dihydrogen phosphate dihydrate (NaH_2_PO_4_·2H_2_O) were provided by Macklin Biochemical Technology Co., Ltd. (Shanghai, China). Ruthenium(III) chloride hexamine complex (Ru(NH_3_)_6_Cl_3_) and ruthenium(II) tris(bipyridine) chloride hexahydrate (Ru(bpy)_3_Cl_2_·6H_2_O) were purchased from Sigma-Aldrich (Shanghai, China). Pasteurized milk was obtained from a local market (Shuangfeng brand). All of the above chemical reagents were of analytical grade and used without further purification. The water used in the experiments was ultrapure water (18.2 MΩ·cm). Indium tin oxide (ITO) conductive glass (sheet resistance < 15 Ω/sq, ITO layer thickness 100 ± 20 nm) was obtained from Zhuhai Kaiwei Optoelectronic Technology Co., Ltd. (Zhuhai, China). The ITO conductive glass was pretreated before use. Specifically, the ITO glass was ultrasonically cleaned in 1 M NaOH solution for 1 h, followed by sequential ultrasonic treatment with acetone, ethanol, and deionized water for 0.5 h each. Finally, the glass was dried by nitrogen blowing.

### 2.2. Measurements and Instrumentation

The microstructure of VMSF was characterized using a Hitachi HT7700 transmission electron microscope (TEM, Tokyo, Japan) at an accelerating voltage of 100 kV. TEM sample preparation involved scraping VMSF from the ITO glass substrate with a blade, dispersing it in ethanol via ultrasonic treatment, and dropping the dispersion onto a copper grid. After the solvent evaporated naturally, the samples were characterized. The VMSF thickness was investigated with a Hitachi SU8010 scanning electron microscope (SEM, Tokyo, Japan) at a 5 kV accelerating voltage. SEM sample preparation included scratching a grid-like pattern onto the electrode surface with a glass cutter, cutting the fractured surface, and fixing the cross-section sample for analysis after gold coating. Electrochemical testing was conducted using an Autolab PGSTAT302N electrochemical workstation (Metrohm, Herisau, Switzerland), where cyclic voltammetry (CV) and differential pulse voltammetry (DPV) were performed in a standard three-electrode system. Specifically, an Ag/AgCl electrode with a saturated KCl solution as the reference electrode, and a standard potential of +0.197 V relative to the standard hydrogen electrode (SHE), was used as the reference electrode. Platinum wire or sheet (1 cm × 1 cm) acted as the counter electrode, and ITO or VMSF-modified ITO electrode (effective area 0.5 × 1 cm^2^) was the working electrode. The employed electrolyte was 0.01 M phosphate buffer solution (PBS, pH 7). Electrochemiluminescence (ECL) testing was carried out using self-constructed ECL analysis platform in the laboratory, where ECL was triggered by CV scanning. The CV scan potential range was 0–1.4 V, with a scan rate of 100 mV/s, and the photomultiplier tube (PMT) working voltage was set to 400 V.

### 2.3. Preparation of VMSF/ITO Electrode

The VMSF was rapidly grown on the pretreated ITO electrode using an electrochemical-assisted self-assembly method (EASA) [54,55]. The precursor solution was prepared by adding 2.833 g of TEOS and 1.585 g of CTAB to a mixture of 20 mL ethanol and 20 mL sodium nitrate solution (0.1 M, pH 2.6), then stirring magnetically at room temperature for 2.5 h. VMSF film growth was achieved by applying a constant current density (−350 μA/cm^2^) on the ITO electrode for 10 s, followed by rinsing the electrode with ultrapure water and drying it with nitrogen. The electrode was then heat-treated at 120 °C for 12 h to complete the aging process, resulting in an electrode containing surfactant micelles (SMs), labeled SM@VMSF/ITO. Finally, the electrode was immersed in a 0.1 M HCl-ethanol solution and stirred for 5 min to remove the micelles, yielding the VMSF/ITO electrode with open nanochannels.

### 2.4. Detection of ALP

Different concentrations of ALP solutions (0.01, 0.1, 5, 10, 15, 20, 30 U/L) were incubated with 1 mM diphenyl iodinium salt (DPP) in PBS buffer (0.01 M, pH 7) at 37 °C for 1 h. After the reaction mixture was cooled to room temperature, the ECL signal was detected using the VMSF/ITO electrode. To evaluate real application of the constructed ECL sensor, pasteurized milk samples were selected as complex matrices and ALP analysis was performed using standard addition method. Specifically, pasteurized milk with spiked ALP was diluted 20 times with PBS buffer (0.01 M, pH 7). Then, the obtained samples were detected after incubation with DPP using the same process. The testing electrolyte for ECL measurement was PBS buffer (0.01 M, pH 7) containing Ru(bpy)_3_^2^^+^ (10 μM) and tripropylamine (TPA, 3 mM).

## 3. Results and Discussion

### 3.1. Characterization of VMSF-Modified Electrode

As illustrated in Figure 1, a homogenous ECL sensor for detecting ALP was developed by using VMSF-modified indium tin oxide (ITO) as the working electrode. This sensor was based on the enrichment of the Ru(bpy)_3_^2^^+^ probe by VMSF and the quenching effect of ALP enzymatic products (Phe) on the ECL signal. For all ECL measurements, TPA was used as a co-reactant for Ru(bpy)_3_^2^^+^.

**Figure 2:**
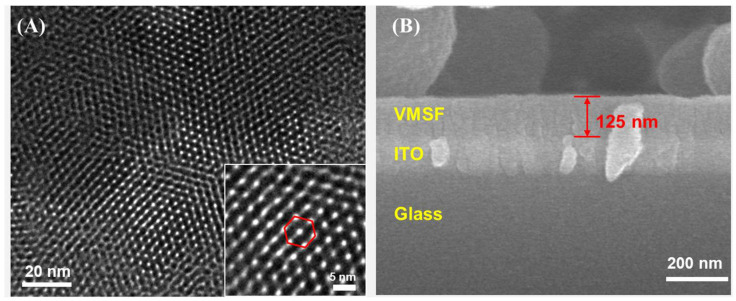
(**A**) Top-view TEM image and (**B**) cross-sectional SEM image of VMSF. The inset in (**A**) shows an enlarged TEM image and the graphic represents the arrangement of nanochannels of VMS F in a hexagonal structure.

The morphology and structure of VMSF were characterized by TEM and SEM, with the results shown in Figure 2. The top-view TEM image in Figure 2A showed that the VMSF channels were arranged in a hexagonal pattern with no visible structural defects, and the pore size was approximately 2–3 nm. The SEM cross-sectional image in Figure 2B revealed the distinct three-layer structure, consisting of a glass substrate, an ITO conductive layer, and a VMSF-modified layer, from bottom to top. The VMSF layer was uniform, approximately 125 nm thick.

**Figure 3:**
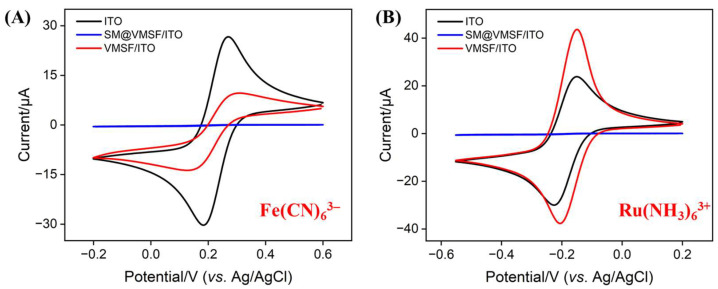
CV curves obtained on bare ITO, SM@VMSF/ITO, and VMSF/ITO electrodes in 0.5 mM (**A**) K_3_Fe(CN)_6_ or (**B**) Ru(NH_3_)_6_Cl_3_ solution. The electrolyte solution was 0.05 M KHP, pH = 7.

To assess the structural integrity and charge-selective permeability of the VMSF, Fe(CN)_6_;^3^^−^ and Ru(NH_3_)_6_;^3^^+^ were used as electrochemical probes, and the electrochemical behavior of the electrodes was studied using CV. As shown in Figure 3, for the SM@VMSF/ITO electrode with surfactant micelle-sealed nanochannels, the hydrophobic SM barrier blocked the transport of Ru(NH_3_)_6_;^3^^+^ cations and Fe(CN)_6_;^3^^−^ anions, preventing redox reactions and leading to almost undetectable Faradaic current signals. This confirmed the structural integrity of the VMSF [56,57]. After removing the SM, the VMSF/ITO electrode with open nanochannels showed a significant enhancement in the redox current for Ru(NH_3_)_6_;^3^^+^ compared to the bare ITO electrode, while the current for Fe(CN)_6_;^3^^−^ was greatly weakened, indicating the charge-selective permeability of VMSF. It electrostatically adsorbed cationic probes while repelling anionic probes, due to the negatively charged surface generated by the ionization of silanol groups on the VMSF surface [58]. This property may enable enrichment of positively charged ECL emitters.

### 3.2. Feasibility Study and Condition Optimization for ALP Detection

**Figure 4:**
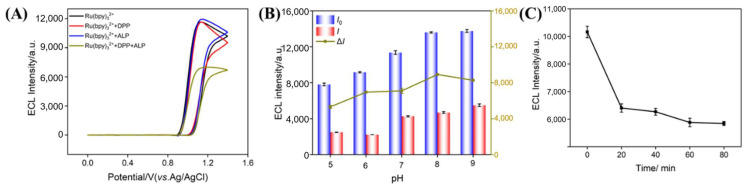
(**A**) ECL response curve of VMSF/ITO in different solutions. Effect of pH (**B**) and incubation time (**C**) of ALP and DPP on the ECL signal, where Δ*I* represents the difference in ECL intensity before (*I*_0_) and after (*I*) incubating ALP in the DPP solution.

In this work, a sensor for the ECL detection of ALP was developed based on the quenching effect of enzymatic product, Phe, on the ECL signal determined by VMSF/ITO electrode. As shown in Figure 4A, even at a low Ru(bpy)_3_^2^^+^ concentration (10 μM), the ECL signal of the Ru(bpy)_3_^2^^+^-TPA system on the VMSF/ITO electrode exceeded 12,000 a.u. In contrast, the ECL signal on the bare ITO electrode was very low (less than 110 a.u., as shown in Figure S1 in the Supporting Information, SI). Thus, the ITO electrode cannot serve as a supporting electrode for constructing a signal-off type sensing system. The significantly increased ECL signal on the VMSF/ITO electrode, compared to the bare ITO electrode, was attributed to the negatively charged surface of VMSF. Specifically, VMSF electrostatically adsorbed the positively charged Ru(bpy)_3_^2^^+^. As a result, even at low solution concentrations, it achieved a high local concentration at the electrode surface. Therefore, using the VMSF/ITO electrode as the detection electrode can significantly enhance the initial ECL signal, providing potential for a wide detection linear range for signal-off sensing. To further verify the feasibility of the sensor for detecting ALP, the effects of the ALP substrate DPP, ALP, and an ALP-DPP mixture on the ECL signal of the Ru(bpy)_3_^2^^+^-TPA system were investigated. Amongst these, the ALP and DPP mixture was incubated at 37 °C for 1 h. As shown in Figure 4A, the ECL signal of the solution was measured using the VMSF/ITO electrode following the addition of Ru(bpy)_3_^2^^+^ and TPA. The results showed that neither DPP nor ALP alone had a significant impact on the ECL signal, whereas the ECL signal from the ALP and DPP co-incubation was significantly reduced. This suggested that the decrease in ECL signal was due to ALP catalyzing the hydrolysis of DPP to produce Phe, which acted as a quencher, diminishing the ECL signal of the Ru(bpy)_3_^2^^+^-TPA system.

To optimize the performance for ALP detection, pH of the test solution and the effects of incubation time between ALP and DPP were systematically studied. The influence of solution pH on ALP detection performance involved two main aspects. On one hand, an increase in pH enhanced the deprotonation of the Si-OH groups (p*K*_a_~2) on the VMSF surface, strengthening its negative charge and improving its ability to enrich Ru(bpy)_3_^2^^+^, which lead to an increased ECL signal. On the other hand, alkaline conditions were more favorable for ALP catalytic activity, promoting substrate hydrolysis to generate more phenol, thus enhancing the quenching efficiency. Figure 4B showed the change in ECL signal before and after adding ALP under different pH conditions, with the highest quenching efficiency observed at pH 8. Considering that excessively high pH values may affect the long-term stability of the VMSF, pH 7 was selected for further investigation. Additionally, Figure 4C showed the optimization of the incubation time for ALP and DPP. The results indicated that the ECL signal stabilized after 60 min of incubation, suggesting that the ALP-catalyzed reaction with DPP had reached equilibrium. Thus, an incubation time of 60 min for ALP and DPP was selected for subsequent studies.

### 3.3. Mechanism of ECL Sensor for ALP Detection

**Figure 5:**
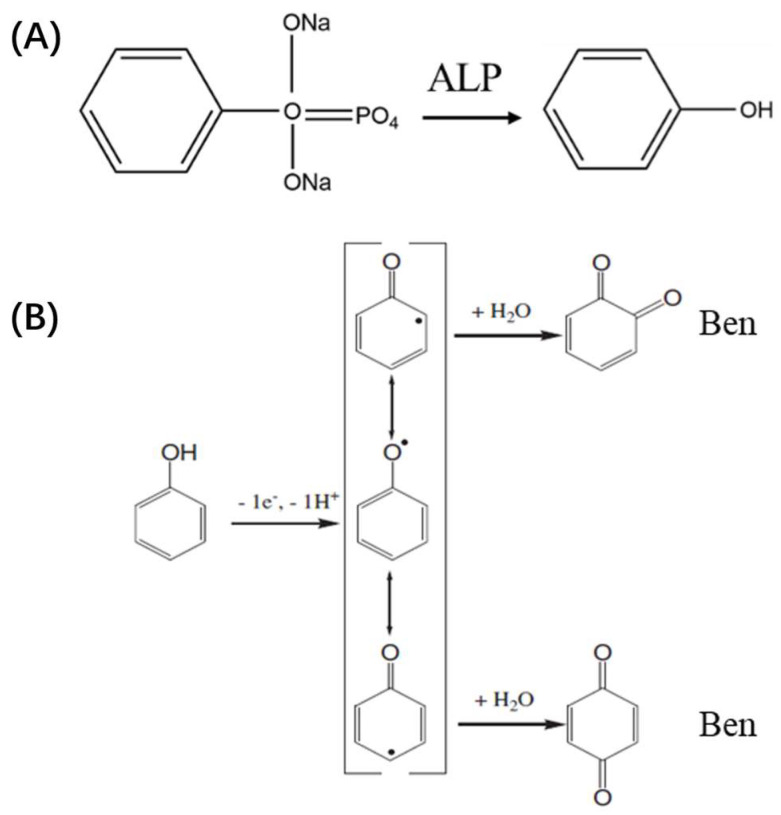
(**A**) Schematic representation of the reaction in which ALP catalyzes the hydrolysis of the substrate DPP to form Phe. (**B**) Schematic representation of the oxidation of Phe at the electrode followed by reaction with H_2_O.

In this work, VMSF was grown on the ITO surface to significantly enhance the ECL signal of the Ru(bpy)_3_^2^^+^ probe system. The detection principle for ALP was illustrated in Figure 1B,C. In the Ru(bpy)_3_^2^^+^/TPA system, the Ru(bpy)_3_^2^^+^ emitter was oxidized to the strong oxidant Ru(bpy)_3_^3^^+^ on the electrode surface, while TPA was oxidized to generate the cationic radical (TPA•^+^). The TPA•^+^ rapidly lost a proton to form the highly reducible radical TPA•, which subsequently reacts with Ru(bpy)_3_^3^^+^ to produce the excited-state (Ru(bpy)_3_^2^^+^*). This excited state then returned to the ground state, emitting light. In the presence of ALP, the enzyme catalyzes the hydrolysis of the substrate DPP to produce phenol (Phe) (Figure 5A) [52]. Phe was oxidized at the electrode, losing one electron and one proton, thereby generating three unstable phenoxy radical intermediates. These intermediates ultimately react with water to form ortho-quinone and para-quinone, referred to as quinones, Ben (Figure 5B) [53].

Ben quenched the ECL signal of the Ru(bpy)_3_^2^^+^/TPA system via two distinct mechanisms: radical intermediate quenching and excited-state quenching. The former occurs when the oxidizing Ben undergoes redox reactions with the reducing TPA• radical on the electrode surface (Equation (1)) [59]. The latter arises due to the significant overlap between the emission spectrum of Ru(bpy)_3_^2^^+^* and the absorption spectrum of Ben, facilitating Förster-type energy transfer between the two species (Equation (2)) [60]. Both quenching mechanisms lead to a reduction in the concentrations of TPA• and Ru(bpy)_3_^2^^+^*, resulting in the decrease of the ECL signal in the Ru(bpy)_3_^2^^+^/TPA system. Given that DPP was present in excess within the reaction system, the amount of Phe generated by ALP activity correlates directly with the ALP concentration, thus the extent of ECL signal quenching was proportional to ALP levels. This mechanism formed the basis for ALP detection.

TPA^•^ + Ben → Pr_2_NC^+^HCH_2_CH_3_ (P_1_) + Reduction product of Ben (P_2_) (1)

Ru(bpy)_3_^2+*^+ Ben → Ru(bpy)_3_^2^^+^ + Ben^*^ (2)

### 3.4. ECL Detection of ALP

**Figure 6:**
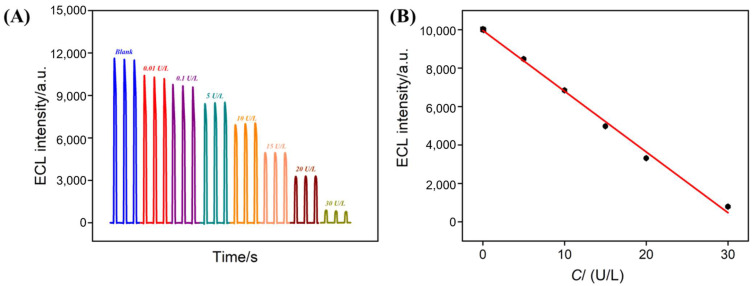
The ECL response curve (**A**) and the corresponding linear relationship (**B**) for ALP detection in the range of 0.01 U/L to 30 U/L using VMSF/ITO in a solution containing 3 mM TPA, 10 μM Ru(bpy)_3_^2^^+^, and 1 mM DPP.

Based on the mechanism in which ALP catalyzes the conversion of DPP to Phe, thereby quenching the ECL signal of the Ru(bpy)_3_^2^^+^-TPA system, ALP can be quantified. The VMSF/ITO electrode was used for ECL detection of ALP. Figure 6A showed the ECL curves measured in the Ru(bpy)_3_^2^^+^-TPA solution after incubating different concentrations of ALP with excess DPP. The ECL signal intensity decreased as the ALP concentration increased. As shown in Figure 6B, when the ALP concentration was in the range of 0.01–30 U/L, the ECL intensity (I) exhibited a good linear relationship with the ALP concentration (C), and the fitting equation was I_ECL_ = −316.1 C + 9961 (R^2^ = 0.998). The limit of detection (LOD) was calculated to be 0.008 U/L based on a three-fold signal-to-noise ratio (S/N = 3).

The LOD and the low concentration within the linear range were lower than the tolerance level of pasteurized milk. This allows for the detection of milk samples with appropriate dilution factors (e.g., diluted by a factor of 20), thereby minimizing the influence of complex sample matrices and enhancing the reliability of detection. Comparison of the ALP detection performance using various ECL methods is shown in Table S1 (SI). The LOD based on signal-off detection using VMSF/ITO was lower than the signal-off detection using tris(1,10-phenanthroline)ruthenium(II) [Ru(phen)_3_^2+^] and tetrahedral chalcogenide nanoclusters of [Cd_32_S_14_(SC_6_H_5_)_38_]^2−^ complex nanocluster-modified glassy carbon electrodes (CdS-Ru/GCE) [29], or luminol-doped silica nanoparticle-modified GCE (luminol-SiNPs/GCE) [61], CdSe nanoparticle-modified GCE (CdSe NPs/GCE) [62], or signal-on detection using Ru(bpy)_3_^2+^ encapsulated zeolite imidazole metal organic framework (Ru(bpy)_3_^2+^@ZIF-90) [63]. However, it was higher than homogeneous signal-on sensing based on click chemistry-triggered branched hybridization chain reaction [64], or turn-on sensing using CsPbBr_3_ perovskite quantum dots modified GCE (CsPbBr_3_ perovskite QDs/GCE) [65], or signal-off sensing using Cu-doped TiO_2_ oxygen vacancy and Au@SiO_2_ nanomembrane modified GCE (Cu-TiO_2_/Au@SiO_2_-NM/GCE) [66]. Owing to the convenient growth of VMSF and the enhanced ECL signal even at a low concentration of emitter, the constructed sensor offers the advantages of simple preparation, high detection sensitivity, and low cost.

### 3.5. Selectivity of the Constructed ECL Sensors

**Figure 7:**
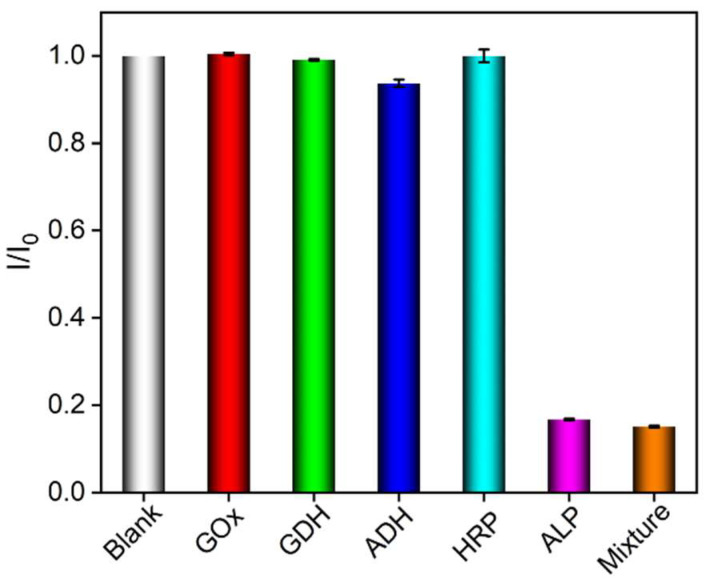
The ratio of ECL intensity obtained on VMSF/ITO electrode in absence (blank) or presence 0.02 U/L ALP or 1 U/L of other species to a PBS solution containing 3 mM TPA, 10 μM Ru(bpy)_3_^2^^+^, and 1 mM DPP.

The selectivity of the sensor was investigated with the ECL signal of the Ru(bpy)_3_^2^^+^-TPA system by incubating other enzymes with DPP. Figure 7 shows the change in the ratio of ECL signal (*I*/*I*_0_) before (*I*_0_) and after (*I*) adding the target or interfering species. “Blank” in the figure refers to the ECL signal change ratio in the absence of added ALP or potential interfering substances, with a value of 1. It can be observed that the ECL signal only significantly changed in presence of ALP or ALP-containing mixtures. Glucose dehydrogenase (GDH), alcohol dehydrogenase (ADH), horseradish peroxidase (HRP), or glucose oxidase (GOX) did not cause remarkable signal change even at 50-fold concentrations of that of ALP. These results indicated a high selectivity of the constructed sensor.

### 3.6. Real Sample Analysis

To further investigate the accuracy of the constructed quenching-type sensor for detecting ALP in complex real samples, pasteurized milk was chosen as the real sample matrix and ALP was detected using the standard addition method. As shown in Table 1, the milk was diluted 20 times with PBS (0.01 M, pH = 7) satisfactory recovery rates (98.4–107%) and low relative standard deviation (RSD, <4.7%) for three parallel measurements were obtained.

## 4. Conclusions

In summary, a nanochannel-modified electrode was employed for sensitive detection of ALP. The VMSF can electrostatically enrich the cationic ECL emitter Ru(bpy)_3_^2^^+^, enhancing the original ECL signal of the electrode. In the presence of ALP, it catalyzed the reaction of its substrate DPP to produce Phe, which acted as the quencher for the ECL signal of the Ru(bpy)_3_^2^^+^-TPA system. Thus, an ECL quenching-type sensor was developed for highly sensitive detection of ALP. Combining the excellent anti-contamination and anti-interference properties of VMSF, the constructed sensor was successfully applied for ALP detection in milk.

## Figures and Tables

**Table 1:** Determination of ALP in pasteurized milk by the fabricated sensor using a standard addition method.

Sample	Added (U/L)	Detected (U/L)	Recovery (%)	RSD (%, n = 3)
Pasteurized milk ^a^	0.100	0.107	107	4.7
	1.00	0.962	96.2	2.9
	5.00	4.92	98.4	3.6

^a^ The sample was diluted 20 times with PBS (0.01 M, pH = 7). The concentration was obtained after dilution.

## Data Availability

The data presented in this study are available on request from the corresponding author.

## Supplementary Materials

The following supporting information can be downloaded at: https://www.mdpi.com/article/10.3390/bios15060377/s1, Figure S1: ECL intensity obtained on bare ITO electrode in 0.01 M PBS (pH 7) containing 3 mM TPA and 10 μM Ru(bpy)_3_^2^^+^; Table S1: Comparison of ALP detection performance using various ECL methods.
