# Supplementary material for: Simple Nanochannel-Modified Electrode for Sensitive Detection of Alkaline Phosphatase Through Electrochemiluminescence Signal Quenching by Enzymatic Reaction
Source: Biosensors (Basel). 2025 Jun 11;15(6):377. doi: 10.3390/bios15060377 (PMC12190674; doi:10.3390/bios15060377)
Supplement: Supplementary file 1 [file biosensors-15-00377-s001.zip › biosensors-3632376-supplementary.pdf]

biosensors

MDPI

Supplementary

Simple Nanochannel-Modified Electrode for Sensitive Detection of Alkaline Phosphatase Through Electrochemiluminescence Signal Quenching by Enzymatic Reaction

Tianjun Ma [1,†], Xuan Luo [2,†], Fengna Xi [2,*] and Nuo Yang [1,*]

1 The First Affiliated Hospital of Guangxi Medical University, Nanning 530021, China; mtj.2008@sr.gxmu.edu.cn
2 School of Chemistry and Chemical Engineering, Zhejiang Sci-Tech University, Hangzhou 310018, China; 202020104138@mails.zstu.edu.cn
* Correspondence: fengnaxi@zstu.edu.cn (F.X.); yangnuo@gxmu.edu.cn (N.Y.)
† These authors contributed equally to this work.

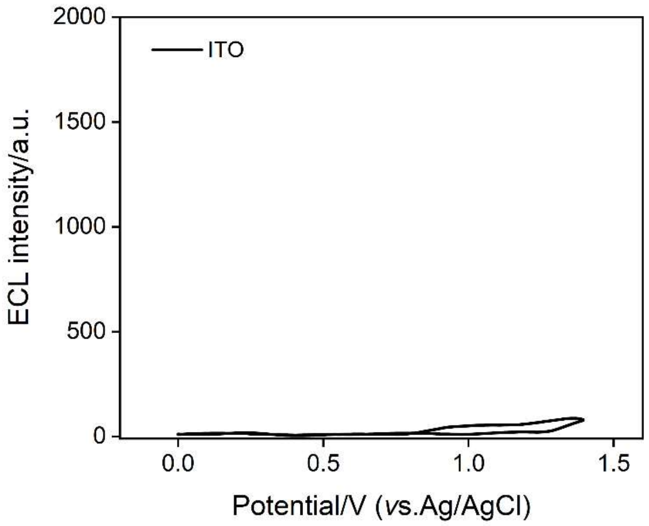

Figure S1. ECL intensity obtained on a bare ITO electrode in 0.01 M PBS (pH 7) containing 3 mM TPA and 10 μM $Ru(bpy)_3^{2+}$.

Table S1. Comparison of ALP detection performance using various ECL methods.

Electrode or system	Sensing mode	Linear range (U/L)	LOD (U/L)	Ref.
CdS-Ru/GCE	signal-off	0.5-10	0.35	[29]
Luminol-SiNPs/GCE	signal-off	5-50	0.8	[61]
CdSe NPs/GCE	signal-off	2-25	2	[62]
$Ru(bpy)_3^{2+}$@ZIF-90	signal-on	0.5-100	0.04	[63]

Click chemistry triggered branched hybridization chain reaction	signal-on	0.002-50	0.0007	[64]
$CsPbBr_3$ perovskite QDs/GCE	signal-on	0.015-0.13	0.007	[65]
Cu-TiO_2/Au@SiO_2-NM/GCE	signal-off	0.005-10	0.005	[66]
VMSF/ITO	signal-off	0.01-30	0.008	**This work**

CdS-Ru: tris(1,10-phenanthroline)ruthenium(II) [Ru(phen)32+] and tetrahedral chalcogenide nanoclusters of [Cd32S14(SC6H5)38]2- complex nanoclusters; GCE: glassy carbon electrode; luminol-SiNPs: luminol-doped silica nanoparticles; NPs: nanoparticles; Ru(bpy)32+@ZIF-90: Ru(bpy)32+ encapsulated zeolite imidazole metal organic framework; QDs: quantum dots; Cu-TiO2/Au@SiO2-NM: Cu-doped TiO2 oxygen vacancy and a Au@SiO2 nanomembrane.
